# 1,1′,1′′-{[4-(3,4-Ethyl­ene­dioxy­thio­phen-2-yl)phen­yl]methane­tri­yl}tris­(1*H*-pyra­zole)

**DOI:** 10.1107/S160053681104253X

**Published:** 2011-10-22

**Authors:** Xiao-Yan Chen, Xiaoping Yang, Bradley J. Holliday

**Affiliations:** a Department of Chemistry and Biochemistry, The University of Texas at Austin, 1 University Station, A5300, Austin, Texas 78712, USA

## Abstract

In the title complex, C_22_H_18_N_6_O_2_S, two of the pyrazole rings are disordered over two sets of sites with ratios of refined occupancies of 0.58 (2):0.42 (2) and 0.517 (12):0.483 (12). The dioxane ring is in a half-chair conformation and the two –CH_2_– groups of this ring are disordered over two sets of sites, the ratio of refined occupancies being 0.855 (19):0.145 (19). The essentially planar thio­phene ring [largest deviation = 0.0444 (2) Å] forms a dihedral angle of 19.59 (3)° with the benzene ring.

## Related literature

For the preparation and coordination chemistry of tris­(pyrazol­yl)borates and tris­(pyrazol­yl)methanes, see: Trofimenko (1999 ▶); Pettinari & Pettinari (2005 ▶); Reger *et al.* (2000 ▶). For the chemistry of tris­(pyrazol­yl)methane derivatives, see: Humphrey *et al.* (1999 ▶). For a general Stille coupling procedure, see: Sankaran *et al.* (2001 ▶). For similar structures, see: Liddle & Gardinier (2007 ▶).
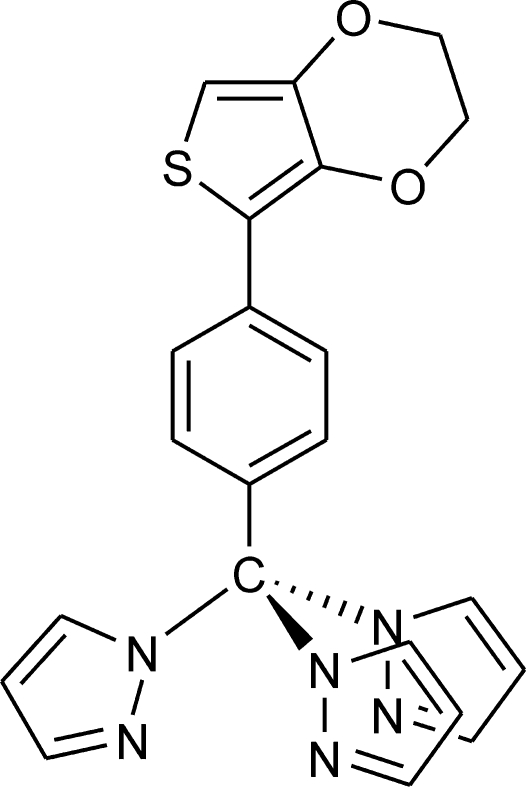


## Experimental

### 

#### Crystal data

C_22_H_18_N_6_O_2_S
*M* *_r_* = 430.48
Triclinic, 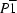

*a* = 7.2356 (14) Å
*b* = 8.1104 (16) Å
*c* = 18.626 (4) Å
α = 95.05 (3)°
β = 99.20 (2)°
γ = 112.14 (3)°
*V* = 986.2 (3) Å^3^
*Z* = 2
Mo *K*α radiation
μ = 0.20 mm^−1^
*T* = 153 K
0.16 × 0.15 × 0.11 mm

#### Data collection

Nonius KappaCCD diffractometer
Absorption correction: gaussian (*SHELXTL*; Sheldrick, 2008 ▶) *T*_min_ = 0.969, *T*_max_ = 0.979
5461 measured reflections
3466 independent reflections
2749 reflections with *I* > 2σ(*I*)
*R*_int_ = 0.025

#### Refinement

*R*[*F* ^2^ > 2σ(*F* ^2^)] = 0.052
*wR*(*F* ^2^) = 0.148
*S* = 1.12
3466 reflections
294 parameters
9 restraints
H-atom parameters constrained
Δρ_max_ = 0.43 e Å^−3^
Δρ_min_ = −0.46 e Å^−3^

### 

Data collection: *COLLECT* (Nonius, 1998 ▶); cell refinement: *COLLECT*; data reduction: *DENZO* and *SCALEPACK* (Otwinowski & Minor, 1997) ▶; program(s) used to solve structure: *SIR97* (Altomare *et al.*, 1999 ▶) within *WinGX* (Farrugia, 1999 ▶); program(s) used to refine structure: *SHELXTL* (Sheldrick, 2008 ▶); molecular graphics: *ORTEP-3* (Farrugia, 1997 ▶) and *POV-RAY* (Persistence of Vision, 2004 ▶); software used to prepare material for publication: *SHELXL97* (Sheldrick, 2008 ▶).

## Supplementary Material

Crystal structure: contains datablock(s) I, global. DOI: 10.1107/S160053681104253X/lh5310sup1.cif

Structure factors: contains datablock(s) I. DOI: 10.1107/S160053681104253X/lh5310Isup2.hkl

Supplementary material file. DOI: 10.1107/S160053681104253X/lh5310Isup3.cml

Additional supplementary materials:  crystallographic information; 3D view; checkCIF report

## Figures and Tables

**Fig. 1.**
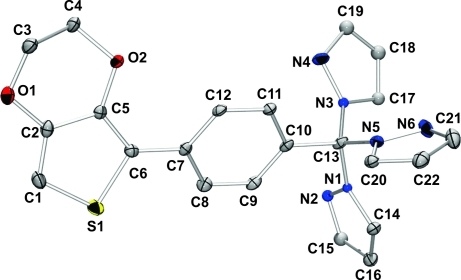
The molecular structure showing 30% probability ellipsoids. Hydrogen atoms are omitted for clarity. The disorder is not shown but disordered atoms can been seen as isotropic spheres.

## supplementary crystallographic information

### Comment

The coordination chemistry of tris(pyrazolyl)borates has been investigated for decades, with variations on the ligand having been widely studied (Trofimenko, 1999; Pettinari & Pettinari, 2005; Reger *et al.*, 2000). However, the isoelectronic tris(pyrazolyl)methanes (Humphrey *et al.*, 1999) have received less attention and there exists a disparity in the chemistry of certain third generation scorpionates (ligands functionalized at the back methine position). Herein, we demonstrate the preparation of a new tris-(pyrazolyl)methane derivative, 1,1',1''-((4-(3,4-(ethylenedioxy)thien-2-yl)phenyl)methanetriyl) tris(1*H*-pyrazole), *via* a Stille coupling (Sankaran *et al.*, 2001). The molecular structure of the title compound is shown in Figure 1. The thiophene ring is essentially planar, with the largest deviation measured as 0.0444 (2) Å. The six-membered dioxane ring exists in a half-chair conformation. The dihedral angle between the benzene and thiophene rings is 19.59 (3) °. This geometry is similar to other scorpionates reported in literature (Liddle & Gardinier, 2007).

### Experimental

The title compound was synthesized from the Stille coupling of 4-(tri(1*H*-pyrazol-1-yl)methyl)phenyl trifluoromethanesulfonate and 2-(tributylstannyl)-3,4-(ethylenedioxy)thiophene. The starting compound, 4-(tri(1*H*pyrazol- 1-yl)methyl)phenol, was prepared by the condensation of 4-(trifluoromethyl)phenol and sodium pyrazol-1-ide in a yield of 58%. Treatment of 4-(tri(1*H*-pyrazol-1-yl)methyl)phenol with trifluoromethane sulfonic acid anhydride in pyridine at 273 K under argon resulted in 4-(tri(1*H*-pyrazol-1-yl)methyl)phenyl trifluoromethanesulfonate in a yield of 97%. The resulting aryl trifluoromethanesulfonate was subsequently coupled with 2-(tributylstannyl)- 3,4-(ethylenedioxy)thiophene in the presence of tetrakis(triphenylphosphine)palladium(0) in 1,4-dioxane to produce the desired compound in a yield of 54%. X-ray quality crystals were grown by slow evaporation of a methanol solution of the title compound.

### Refinement

All H atoms were positioned geometrically and refined using a riding model, with C—H = 0.95 Å and with *U*_iso_(H) = 1.2*U*_eq_(C).

The pyrazole ring containing N3/N4 was disordered by a partial rotation about the C13—N3 bond. The disorder was modeled by assinging the variable *x* to the site occupancy factor of the atoms in one orientation, N3, N4, C19, C18, C17, and (1 - *x*) to the site occupancy factor of the alternate orientation composed of N3A, N4, C19A, C18A, C17A. A common isotropic displacement parameter was refined for the atoms of the ring while geometric restraints were applied throughout the refinement. In this way, the site occupancy factor for atoms N3, N4, C19, C18, C17 refined to 48 (2)%. The pyrazole ring containing N1/N2 was refined in a similar manner. The disordered atoms of the two pyrazole rings remain isotropic.

### Crystal data

**Table:**

C_22_H_18_N_6_O_2_S	*Z* = 2
*M**_r_* = 430.48	*F*(000) = 448
Triclinic, *P*1	*D*_x_ = 1.450 Mg m^−^^3^
Hall symbol: -P 1	Mo *K*α radiation, λ = 0.71073 Å
*a* = 7.2356 (14) Å	Cell parameters from 3640 reflections
*b* = 8.1104 (16) Å	θ = 2.9–30.5°
*c* = 18.626 (4) Å	µ = 0.20 mm^−^^1^
α = 95.05 (3)°	*T* = 153 K
β = 99.20 (2)°	Block, colourless
γ = 112.14 (3)°	0.16 × 0.15 × 0.11 mm
*V* = 986.2 (3) Å^3^	

### Data collection

**Table:**

Nonius KappaCCD diffractometer	3466 independent reflections
Radiation source: fine-focus sealed tube	2749 reflections with *I* > 2σ(*I*)
graphite	*R*_int_ = 0.025
ω scans	θ_max_ = 25.0°, θ_min_ = 3.1°
Absorption correction: gaussian (*SHELXTL*; Sheldrick, 2008)	*h* = −8→8
*T*_min_ = 0.969, *T*_max_ = 0.979	*k* = −8→9
5461 measured reflections	*l* = −22→22

### Refinement

**Table:**

Refinement on *F*^2^	Primary atom site location: structure-invariant direct methods
Least-squares matrix: full	Secondary atom site location: difference Fourier map
*R*[*F*^2^ > 2σ(*F*^2^)] = 0.052	Hydrogen site location: inferred from neighbouring sites
*wR*(*F*^2^) = 0.148	H-atom parameters constrained
*S* = 1.12	*w* = 1/[σ^2^(*F*_o_^2^) + (0.0645*P*)^2^ + 1.0306*P*] where *P* = (*F*_o_^2^ + 2*F*_c_^2^)/3
3466 reflections	(Δ/σ)_max_ < 0.001
294 parameters	Δρ_max_ = 0.43 e Å^−^^3^
9 restraints	Δρ_min_ = −0.46 e Å^−^^3^

### Special details

**Table:**

Geometry. All e.s.d.'s (except the e.s.d. in the dihedral angle between two l.s. planes) are estimated using the full covariance matrix. The cell e.s.d.'s are taken into account individually in the estimation of e.s.d.'s in distances, angles and torsion angles; correlations between e.s.d.'s in cell parameters are only used when they are defined by crystal symmetry. An approximate (isotropic) treatment of cell e.s.d.'s is used for estimating e.s.d.'s involving l.s. planes.
Refinement. Refinement of *F*^2^ against ALL reflections. The weighted *R*-factor *wR* and goodness of fit *S* are based on *F*^2^, conventional *R*-factors *R* are based on *F*, with *F* set to zero for negative *F*^2^. The threshold expression of *F*^2^ > σ(*F*^2^) is used only for calculating *R*-factors(gt) *etc*. and is not relevant to the choice of reflections for refinement. *R*-factors based on *F*^2^ are statistically about twice as large as those based on *F*, and *R*- factors based on ALL data will be even larger.

### Fractional atomic coordinates and isotropic or equivalent isotropic displacement parameters (Å^2^)

**Table:**

	*x*	*y*	*z*	*U*_iso_*/*U*_eq_	Occ. (<1)
S1	0.70254 (12)	0.81579 (11)	0.44453 (4)	0.0321 (2)	
O1	0.5573 (3)	0.7026 (3)	0.63060 (11)	0.0330 (5)	
O2	0.2139 (3)	0.6959 (3)	0.52420 (11)	0.0270 (5)	
N4	−0.3420 (4)	0.5741 (3)	0.20905 (14)	0.0304 (6)	
N5	−0.1013 (3)	0.9898 (3)	0.19286 (12)	0.0211 (5)	
N6	−0.2521 (4)	1.0130 (4)	0.14646 (14)	0.0353 (7)	
C1	0.7421 (5)	0.7770 (4)	0.53348 (17)	0.0316 (7)	
H1	0.8682	0.7828	0.5604	0.038*	
C2	0.5699 (5)	0.7370 (4)	0.56037 (15)	0.0243 (7)	
C3	0.3742 (8)	0.7069 (13)	0.6507 (3)	0.0310 (15)	0.855 (19)
H3A	0.3921	0.8339	0.6635	0.037*	0.855 (19)
H3B	0.3511	0.6484	0.6948	0.037*	0.855 (19)
C4	0.1914 (9)	0.6121 (11)	0.5890 (3)	0.0289 (15)	0.855 (19)
H4A	0.1721	0.4846	0.5768	0.035*	0.855 (19)
H4B	0.0682	0.6137	0.6052	0.035*	0.855 (19)
C3A	0.359 (5)	0.615 (6)	0.6379 (19)	0.039 (9)	0.145 (19)
H3C	0.3554	0.6129	0.6907	0.047*	0.145 (19)
H3D	0.3040	0.4890	0.6122	0.047*	0.145 (19)
C4A	0.232 (5)	0.709 (7)	0.6058 (15)	0.032 (8)	0.145 (19)
H4C	0.2937	0.8381	0.6288	0.038*	0.145 (19)
H4D	0.0938	0.6549	0.6168	0.038*	0.145 (19)
C5	0.4023 (4)	0.7346 (3)	0.50830 (15)	0.0195 (6)	
C6	0.4478 (4)	0.7732 (4)	0.44105 (15)	0.0210 (6)	
C7	0.3179 (4)	0.7824 (3)	0.37400 (15)	0.0208 (6)	
C8	0.3706 (5)	0.7717 (4)	0.30513 (16)	0.0265 (7)	
H8A	0.4919	0.7548	0.3015	0.032*	
C9	0.2494 (5)	0.7853 (4)	0.24205 (15)	0.0250 (7)	
H9A	0.2896	0.7797	0.1961	0.030*	
C10	0.0697 (4)	0.8068 (3)	0.24590 (15)	0.0213 (6)	
C11	0.0162 (4)	0.8172 (4)	0.31440 (15)	0.0236 (6)	
H11A	−0.1064	0.8321	0.3178	0.028*	
C12	0.1377 (4)	0.8061 (4)	0.37725 (15)	0.0224 (6)	
H12	0.0985	0.8147	0.4233	0.027*	
C13	−0.0677 (4)	0.8237 (4)	0.17826 (15)	0.0234 (7)	
C14	0.1425 (4)	0.9858 (4)	0.09120 (15)	0.0242 (7)	
H14A	0.1559	1.1045	0.1082	0.029*	
N1	0.0423 (14)	0.8309 (8)	0.1170 (4)	0.012 (3)*	0.42 (2)
N2	0.0684 (18)	0.6899 (7)	0.0800 (3)	0.018 (2)*	0.42 (2)
C15	0.180 (2)	0.7485 (7)	0.0295 (4)	0.028 (3)*	0.42 (2)
H15A	0.2229	0.6793	−0.0028	0.033*	0.42 (2)
N1A	0.0096 (11)	0.8381 (6)	0.1101 (3)	0.019 (2)*	0.58 (2)
N2A	−0.0119 (14)	0.6789 (6)	0.0712 (3)	0.0246 (17)*	0.58 (2)
C15A	0.1144 (15)	0.7421 (5)	0.0252 (3)	0.0270 (19)*	0.58 (2)
H15B	0.1313	0.6633	−0.0116	0.032*	0.58 (2)
C16	0.2206 (5)	0.9305 (4)	0.03417 (16)	0.0307 (7)	
H16A	0.2915	1.0069	0.0030	0.037*	
N3	−0.2502 (8)	0.6548 (7)	0.1554 (3)	0.0144 (16)*	0.483 (12)
C17	−0.3761 (10)	0.6025 (8)	0.0880 (3)	0.0231 (19)*	0.483 (12)
H17A	−0.3440	0.6460	0.0439	0.028*	0.483 (12)
C18	−0.5556 (11)	0.4771 (9)	0.0952 (4)	0.030 (2)*	0.483 (12)
H18A	−0.6694	0.4060	0.0568	0.036*	0.483 (12)
C19	−0.5387 (17)	0.4739 (15)	0.1706 (5)	0.029 (4)*	0.483 (12)
H19A	−0.6490	0.4107	0.1927	0.035*	0.483 (12)
N3A	−0.2799 (7)	0.6848 (8)	0.1619 (3)	0.0165 (16)*	0.517 (12)
C17A	−0.4363 (10)	0.6444 (9)	0.1030 (4)	0.036 (2)*	0.517 (12)
H17B	−0.4336	0.7065	0.0620	0.043*	0.517 (12)
C18A	−0.5959 (10)	0.5005 (7)	0.1130 (4)	0.0243 (17)*	0.517 (12)
H18B	−0.7279	0.4424	0.0821	0.029*	0.517 (12)
C19A	−0.5217 (15)	0.4570 (14)	0.1794 (4)	0.026 (3)*	0.517 (12)
H19B	−0.5945	0.3545	0.1999	0.032*	0.517 (12)
C20	0.0224 (4)	1.1452 (4)	0.24024 (15)	0.0228 (6)	
H20A	0.1386	1.1618	0.2767	0.027*	
C21	−0.2231 (6)	1.1819 (5)	0.16671 (19)	0.0413 (9)	
H21A	−0.3072	1.2373	0.1445	0.050*	
C22	−0.0533 (5)	1.2711 (4)	0.22500 (18)	0.0331 (8)	
H22A	−0.0025	1.3930	0.2486	0.040*	

### Atomic displacement parameters (Å^2^)

**Table:**

	*U*^11^	*U*^22^	*U*^33^	*U*^12^	*U*^13^	*U*^23^
S1	0.0269 (4)	0.0472 (5)	0.0283 (4)	0.0194 (4)	0.0107 (3)	0.0060 (3)
O1	0.0304 (12)	0.0432 (13)	0.0218 (11)	0.0125 (10)	−0.0015 (9)	0.0090 (9)
O2	0.0222 (11)	0.0382 (12)	0.0208 (11)	0.0095 (9)	0.0063 (8)	0.0138 (9)
N4	0.0240 (14)	0.0293 (14)	0.0288 (14)	−0.0008 (11)	0.0073 (11)	0.0075 (11)
N5	0.0183 (12)	0.0281 (13)	0.0142 (12)	0.0065 (10)	0.0032 (10)	0.0032 (10)
N6	0.0291 (15)	0.0603 (19)	0.0214 (14)	0.0252 (14)	0.0003 (11)	0.0054 (13)
C1	0.0266 (17)	0.0429 (18)	0.0266 (17)	0.0178 (14)	0.0005 (13)	0.0041 (14)
C2	0.0296 (17)	0.0216 (14)	0.0192 (15)	0.0102 (12)	−0.0007 (12)	0.0010 (11)
C3	0.031 (3)	0.036 (4)	0.022 (2)	0.009 (3)	0.006 (2)	0.010 (2)
C4	0.030 (3)	0.035 (3)	0.021 (3)	0.011 (3)	0.005 (2)	0.012 (3)
C3A	0.045 (17)	0.014 (16)	0.042 (17)	−0.007 (14)	0.013 (12)	0.007 (13)
C4A	0.025 (14)	0.04 (2)	0.020 (14)	0.005 (14)	0.002 (11)	−0.004 (13)
C5	0.0222 (15)	0.0149 (13)	0.0209 (15)	0.0073 (11)	0.0035 (12)	0.0021 (11)
C6	0.0219 (15)	0.0197 (13)	0.0239 (15)	0.0102 (11)	0.0077 (12)	0.0023 (11)
C7	0.0262 (16)	0.0152 (13)	0.0196 (14)	0.0067 (12)	0.0049 (12)	0.0032 (11)
C8	0.0320 (17)	0.0284 (15)	0.0236 (15)	0.0157 (13)	0.0100 (13)	0.0026 (12)
C9	0.0371 (18)	0.0235 (15)	0.0154 (14)	0.0113 (13)	0.0106 (13)	0.0023 (11)
C10	0.0267 (16)	0.0151 (13)	0.0180 (14)	0.0033 (11)	0.0055 (12)	0.0035 (11)
C11	0.0236 (15)	0.0301 (15)	0.0195 (15)	0.0107 (12)	0.0085 (12)	0.0088 (12)
C12	0.0256 (16)	0.0262 (15)	0.0161 (14)	0.0082 (12)	0.0093 (12)	0.0073 (11)
C13	0.0281 (16)	0.0201 (14)	0.0134 (14)	0.0005 (12)	0.0032 (12)	0.0030 (11)
C14	0.0272 (16)	0.0226 (14)	0.0236 (15)	0.0090 (12)	0.0089 (12)	0.0065 (12)
C16	0.0335 (18)	0.0406 (18)	0.0229 (16)	0.0177 (15)	0.0096 (14)	0.0099 (13)
C20	0.0225 (15)	0.0240 (15)	0.0204 (14)	0.0068 (12)	0.0058 (12)	0.0045 (11)
C21	0.047 (2)	0.065 (2)	0.0311 (18)	0.0402 (19)	0.0115 (16)	0.0143 (17)
C22	0.043 (2)	0.0348 (17)	0.0298 (17)	0.0202 (15)	0.0160 (15)	0.0096 (14)

### Geometric parameters (Å, °)

**Table:**

S1—C1	1.713 (3)	C10—C13	1.526 (4)
S1—C6	1.732 (3)	C11—C12	1.378 (4)
O1—C2	1.371 (4)	C11—H11A	0.9500
O1—C3A	1.37 (3)	C12—H12	0.9500
O1—C3	1.445 (5)	C13—N1A	1.463 (4)
O2—C5	1.366 (3)	C13—N3	1.468 (4)
O2—C4	1.439 (4)	C13—N3A	1.485 (4)
O2—C4A	1.50 (3)	C13—N1	1.486 (5)
N4—C19A	1.283 (10)	C14—N1A	1.341 (5)
N4—N3A	1.320 (5)	C14—N1	1.366 (6)
N4—N3	1.366 (6)	C14—C16	1.396 (3)
N4—C19	1.378 (11)	C14—H14A	0.9500
N5—N6	1.362 (3)	N1—N2	1.372 (5)
N5—C20	1.367 (4)	N2—C15	1.340 (6)
N5—C13	1.466 (4)	C15—C16	1.385 (5)
N6—C21	1.316 (5)	C15—H15A	0.9500
C1—C2	1.354 (4)	N1A—N2A	1.366 (5)
C1—H1	0.9500	N2A—C15A	1.344 (6)
C2—C5	1.420 (4)	C15A—C16	1.408 (5)
C3—C4	1.501 (11)	C15A—H15B	0.9500
C3—H3A	0.9900	C16—H16A	0.9500
C3—H3B	0.9900	N3—C17	1.355 (5)
C4—H4A	0.9900	C17—C18	1.350 (7)
C4—H4B	0.9900	C17—H17A	0.9500
C3A—C4A	1.49 (7)	C18—C19	1.394 (7)
C3A—H3C	0.9900	C18—H18A	0.9500
C3A—H3D	0.9900	C19—H19A	0.9500
C4A—H4C	0.9900	N3A—C17A	1.361 (5)
C4A—H4D	0.9900	C17A—C18A	1.350 (6)
C5—C6	1.378 (4)	C17A—H17B	0.9500
C6—C7	1.463 (4)	C18A—C19A	1.396 (7)
C7—C12	1.398 (4)	C18A—H18B	0.9500
C7—C8	1.400 (4)	C19A—H19B	0.9500
C8—C9	1.388 (4)	C20—C22	1.359 (4)
C8—H8A	0.9500	C20—H20A	0.9500
C9—C10	1.387 (4)	C21—C22	1.406 (5)
C9—H9A	0.9500	C21—H21A	0.9500
C10—C11	1.396 (4)	C22—H22A	0.9500
			
C1—S1—C6	93.25 (15)	N1A—C13—N3	100.5 (4)
C2—O1—C3A	112.4 (13)	N5—C13—N3	116.8 (4)
C2—O1—C3	112.3 (3)	N1A—C13—N3A	108.9 (4)
C5—O2—C4	112.3 (3)	N5—C13—N3A	101.5 (4)
C5—O2—C4A	109.3 (11)	N5—C13—N1	110.7 (4)
C19A—N4—N3A	108.0 (4)	N3—C13—N1	104.0 (4)
C19A—N4—N3	109.2 (4)	N3A—C13—N1	114.7 (4)
N3A—N4—C19	98.7 (5)	N1A—C13—C10	117.0 (4)
N3—N4—C19	101.9 (4)	N5—C13—C10	109.3 (2)
N6—N5—C20	111.7 (2)	N3—C13—C10	109.2 (3)
N6—N5—C13	118.9 (2)	N3A—C13—C10	114.4 (3)
C20—N5—C13	128.3 (2)	N1—C13—C10	106.2 (5)
C21—N6—N5	104.4 (3)	N1A—C14—C16	108.1 (3)
C2—C1—S1	111.0 (2)	N1—C14—C16	104.5 (3)
C2—C1—H1	124.5	N1A—C14—H14A	123.1
S1—C1—H1	124.5	N1—C14—H14A	127.8
C1—C2—O1	123.9 (3)	C16—C14—H14A	127.8
C1—C2—C5	113.0 (3)	C14—N1—N2	109.4 (4)
O1—C2—C5	123.1 (3)	C14—N1—C13	123.8 (4)
O1—C3—C4	111.5 (6)	N2—N1—C13	126.8 (6)
O1—C3—H3A	109.3	C15—N2—N1	109.8 (5)
C4—C3—H3A	109.3	N2—C15—C16	105.8 (5)
O1—C3—H3B	109.3	N2—C15—H15A	127.1
C4—C3—H3B	109.3	C16—C15—H15A	127.1
H3A—C3—H3B	108.0	C14—N1A—N2A	114.6 (3)
O2—C4—C3	111.7 (6)	C14—N1A—C13	127.5 (4)
O2—C4—H4A	109.3	N2A—N1A—C13	115.4 (4)
C3—C4—H4A	109.3	C15A—N2A—N1A	99.8 (4)
O2—C4—H4B	109.3	N2A—C15A—C16	116.9 (5)
C3—C4—H4B	109.3	N2A—C15A—H15B	121.5
H4A—C4—H4B	108.0	C16—C15A—H15B	121.5
O1—C3A—C4A	110 (3)	C15—C16—C14	110.3 (4)
O1—C3A—H3C	109.7	C14—C16—C15A	100.5 (4)
C4A—C3A—H3C	109.7	C14—C16—H16A	124.9
O1—C3A—H3D	109.7	C15A—C16—H16A	132.0
C4A—C3A—H3D	109.7	C17—N3—N4	113.1 (4)
H3C—C3A—H3D	108.2	C17—N3—C13	125.0 (4)
C3A—C4A—O2	111 (4)	N4—N3—C13	118.0 (4)
C3A—C4A—H4C	109.4	C18—C17—N3	107.2 (5)
O2—C4A—H4C	109.4	C18—C17—H17A	126.4
C3A—C4A—H4D	109.4	N3—C17—H17A	126.4
O2—C4A—H4D	109.4	C17—C18—C19	105.6 (7)
H4C—C4A—H4D	108.0	C17—C18—H18A	127.2
O2—C5—C6	123.9 (3)	C19—C18—H18A	127.2
O2—C5—C2	122.4 (2)	N4—C19—C18	111.4 (8)
C6—C5—C2	113.7 (3)	N4—C19—H19A	124.3
C5—C6—C7	130.1 (3)	C18—C19—H19A	124.3
C5—C6—S1	109.0 (2)	N4—N3A—C17A	108.9 (4)
C7—C6—S1	120.9 (2)	N4—N3A—C13	120.0 (3)
C12—C7—C8	117.7 (3)	C17A—N3A—C13	131.1 (4)
C12—C7—C6	120.5 (2)	C18A—C17A—N3A	108.0 (5)
C8—C7—C6	121.8 (3)	C18A—C17A—H17B	126.0
C9—C8—C7	121.5 (3)	N3A—C17A—H17B	126.0
C9—C8—H8A	119.3	C17A—C18A—C19A	103.9 (6)
C7—C8—H8A	119.3	C17A—C18A—H18B	128.1
C10—C9—C8	120.2 (3)	C19A—C18A—H18B	128.1
C10—C9—H9A	119.9	N4—C19A—C18A	110.8 (7)
C8—C9—H9A	119.9	N4—C19A—H19B	124.6
C9—C10—C11	118.6 (3)	C18A—C19A—H19B	124.6
C9—C10—C13	122.2 (2)	C22—C20—N5	106.7 (3)
C11—C10—C13	119.2 (3)	C22—C20—H20A	126.7
C12—C11—C10	121.3 (3)	N5—C20—H20A	126.7
C12—C11—H11A	119.4	N6—C21—C22	112.3 (3)
C10—C11—H11A	119.4	N6—C21—H21A	123.9
C11—C12—C7	120.7 (3)	C22—C21—H21A	123.9
C11—C12—H12	119.6	C20—C22—C21	105.0 (3)
C7—C12—H12	119.6	C20—C22—H22A	127.5
N1A—C13—N5	104.1 (3)	C21—C22—H22A	127.5
			
C20—N5—N6—C21	−1.4 (3)	C10—C13—N1—N2	73.6 (8)
C13—N5—N6—C21	−170.4 (2)	C14—N1—N2—C15	−1.1 (10)
C6—S1—C1—C2	−1.3 (3)	C13—N1—N2—C15	−178.4 (8)
S1—C1—C2—O1	−178.9 (2)	N1—N2—C15—C16	−1.5 (10)
S1—C1—C2—C5	0.9 (3)	N1—C14—N1A—N2A	−73.0 (14)
C3A—O1—C2—C1	−162 (2)	C16—C14—N1A—N2A	0.8 (6)
C3—O1—C2—C1	166.0 (5)	N1—C14—N1A—C13	88.3 (16)
C3A—O1—C2—C5	18 (2)	C16—C14—N1A—C13	162.1 (6)
C3—O1—C2—C5	−13.8 (5)	N5—C13—N1A—C14	40.3 (8)
C2—O1—C3—C4	42.9 (8)	N3—C13—N1A—C14	161.6 (7)
C3A—O1—C3—C4	−54 (3)	N3A—C13—N1A—C14	147.9 (7)
C5—O2—C4—C3	45.3 (8)	N1—C13—N1A—C14	−88 (2)
C4A—O2—C4—C3	−45 (2)	C10—C13—N1A—C14	−80.5 (8)
O1—C3—C4—O2	−61.0 (9)	N5—C13—N1A—N2A	−158.6 (5)
C2—O1—C3A—C4A	−49 (4)	N3—C13—N1A—N2A	−37.3 (6)
C3—O1—C3A—C4A	47 (4)	N3A—C13—N1A—N2A	−50.9 (7)
O1—C3A—C4A—O2	66 (5)	N1—C13—N1A—N2A	72.8 (19)
C5—O2—C4A—C3A	−46 (4)	C10—C13—N1A—N2A	80.7 (6)
C4—O2—C4A—C3A	55 (4)	C14—N1A—N2A—C15A	−1.9 (7)
C4—O2—C5—C6	163.5 (4)	C13—N1A—N2A—C15A	−165.5 (6)
C4A—O2—C5—C6	−165 (2)	N1A—N2A—C15A—C16	2.3 (7)
C4—O2—C5—C2	−16.0 (5)	N2—C15—C16—C14	3.5 (8)
C4A—O2—C5—C2	15 (2)	N2—C15—C16—C15A	−57.3 (11)
C1—C2—C5—O2	179.5 (3)	N1A—C14—C16—C15	−15.9 (6)
O1—C2—C5—O2	−0.7 (4)	N1—C14—C16—C15	−4.1 (7)
C1—C2—C5—C6	0.0 (4)	N1A—C14—C16—C15A	0.6 (5)
O1—C2—C5—C6	179.8 (2)	N1—C14—C16—C15A	12.3 (5)
O2—C5—C6—C7	0.0 (5)	N2A—C15A—C16—C15	121.7 (15)
C2—C5—C6—C7	179.4 (3)	N2A—C15A—C16—C14	−1.9 (7)
O2—C5—C6—S1	179.5 (2)	C19A—N4—N3—C17	5.3 (8)
C2—C5—C6—S1	−1.0 (3)	N3A—N4—N3—C17	−83.7 (10)
C1—S1—C6—C5	1.3 (2)	C19—N4—N3—C17	−3.0 (8)
C1—S1—C6—C7	−179.1 (2)	C19A—N4—N3—C13	164.3 (7)
C5—C6—C7—C12	20.3 (4)	N3A—N4—N3—C13	75.3 (8)
S1—C6—C7—C12	−159.3 (2)	C19—N4—N3—C13	156.0 (7)
C5—C6—C7—C8	−161.1 (3)	N1A—C13—N3—C17	−37.6 (8)
S1—C6—C7—C8	19.4 (4)	N5—C13—N3—C17	74.2 (7)
C12—C7—C8—C9	0.4 (4)	N3A—C13—N3—C17	86.6 (13)
C6—C7—C8—C9	−178.3 (2)	N1—C13—N3—C17	−48.1 (8)
C7—C8—C9—C10	−1.1 (4)	C10—C13—N3—C17	−161.2 (6)
C8—C9—C10—C11	0.9 (4)	N1A—C13—N3—N4	166.1 (6)
C8—C9—C10—C13	179.5 (2)	N5—C13—N3—N4	−82.1 (6)
C9—C10—C11—C12	−0.1 (4)	N3A—C13—N3—N4	−69.7 (12)
C13—C10—C11—C12	−178.7 (2)	N1—C13—N3—N4	155.6 (6)
C10—C11—C12—C7	−0.6 (4)	C10—C13—N3—N4	42.6 (7)
C8—C7—C12—C11	0.5 (4)	N4—N3—C17—C18	−2.7 (9)
C6—C7—C12—C11	179.2 (3)	C13—N3—C17—C18	−159.9 (7)
N6—N5—C13—N1A	67.9 (4)	N3—C17—C18—C19	7.0 (10)
C20—N5—C13—N1A	−99.1 (4)	C19A—N4—C19—C18	−126 (4)
N6—N5—C13—N3	−41.8 (4)	N3A—N4—C19—C18	24.6 (9)
C20—N5—C13—N3	151.2 (3)	N3—N4—C19—C18	7.5 (9)
N6—N5—C13—N3A	−45.2 (3)	C17—C18—C19—N4	−9.4 (11)
C20—N5—C13—N3A	147.8 (3)	C19A—N4—N3A—C17A	5.9 (8)
N6—N5—C13—N1	76.9 (5)	N3—N4—N3A—C17A	102.7 (10)
C20—N5—C13—N1	−90.1 (5)	C19—N4—N3A—C17A	0.4 (7)
N6—N5—C13—C10	−166.4 (2)	C19A—N4—N3A—C13	−173.8 (7)
C20—N5—C13—C10	26.6 (4)	N3—N4—N3A—C13	−77.0 (8)
C9—C10—C13—N1A	−7.2 (4)	C19—N4—N3A—C13	−179.3 (7)
C11—C10—C13—N1A	171.4 (3)	N1A—C13—N3A—N4	141.0 (6)
C9—C10—C13—N5	−125.1 (3)	N5—C13—N3A—N4	−109.6 (6)
C11—C10—C13—N5	53.5 (3)	N3—C13—N3A—N4	81.7 (13)
C9—C10—C13—N3	106.0 (4)	N1—C13—N3A—N4	131.0 (7)
C11—C10—C13—N3	−75.4 (4)	C10—C13—N3A—N4	8.0 (8)
C9—C10—C13—N3A	121.9 (4)	N1A—C13—N3A—C17A	−38.7 (9)
C11—C10—C13—N3A	−59.5 (5)	N5—C13—N3A—C17A	70.8 (8)
C9—C10—C13—N1	−5.6 (4)	N3—C13—N3A—C17A	−98.0 (14)
C11—C10—C13—N1	173.0 (3)	N1—C13—N3A—C17A	−48.6 (9)
N1A—C14—N1—N2	112.6 (17)	C10—C13—N3A—C17A	−171.6 (6)
C16—C14—N1—N2	3.2 (7)	N4—N3A—C17A—C18A	−2.4 (8)
N1A—C14—N1—C13	−70.0 (13)	C13—N3A—C17A—C18A	177.2 (7)
C16—C14—N1—C13	−179.4 (7)	N3A—C17A—C18A—C19A	−1.7 (9)
N1A—C13—N1—C14	69.4 (19)	N3A—N4—C19A—C18A	−7.1 (9)
N5—C13—N1—C14	15.3 (10)	N3—N4—C19A—C18A	−25.2 (9)
N3—C13—N1—C14	141.6 (8)	C19—N4—C19A—C18A	23 (3)
N3A—C13—N1—C14	129.4 (8)	C17A—C18A—C19A—N4	5.5 (10)
C10—C13—N1—C14	−103.3 (8)	N6—N5—C20—C22	1.3 (3)
N1A—C13—N1—N2	−114 (2)	C13—N5—C20—C22	169.0 (3)
N5—C13—N1—N2	−167.8 (7)	N5—N6—C21—C22	1.0 (4)
N3—C13—N1—N2	−41.5 (9)	N5—C20—C22—C21	−0.6 (3)
N3A—C13—N1—N2	−53.7 (10)	N6—C21—C22—C20	−0.2 (4)
